# Comparative efficacy and safety of symptomatic therapy and disease-modifying therapy for Alzheimer’s disease: a systematic review and network meta-analysis

**DOI:** 10.3389/fnins.2025.1656906

**Published:** 2025-11-13

**Authors:** Shiyu Liu, Min Zhao, Yuan Liu, Xin Yang, Huayu Yan, Hongcai Xu, Yabo Wu, Yumin Xu

**Affiliations:** 1 Department of Encephalopathy, The First Affiliated Hospital of Henan University of Chinese Medicine, Zhengzhou, China; 2 The First Clinical Medical College of Henan University of Chinese Medicine, Zhengzhou, China; 3 Collaborative Innovation Center of Prevention and Treatment of Major Diseases by Chinese and Western Medicine, Zhengzhou, China

**Keywords:** Alzheimer’s disease, disease-modifying therapy, network meta-analysis, aducanumab, lecanemab, donanemab

## Abstract

**Background:**

The management of Alzheimer’s disease has shifted toward disease-modifying therapies aimed at delaying disease progression rather than focusing solely on symptomatic treatment. This study summarizes the latest evidence regarding the benefits and harms of anti-Alzheimer’s disease drugs.

**Methods:**

We conducted a comprehensive review of randomized controlled trials from PubMed, Embase, Cochrane Library, Web of Science databases, and other sources up to April 2025. Two researchers independently reviewed the literature and analyzed the data. A network meta-analysis was performed using Review Manager version 5.3 and Stata version 18.0 to calculate mean differences (MDs) and 95% confidence intervals (CIs) for direct and indirect comparisons. Treatment efficacy was evaluated using the Surface Under the Cumulative Ranking Curve (SUCRA). Bias was assessed using the Revised Cochrane Risk of Bias Tool version 2.0, and publication bias was analyzed with funnel plots.

**Results:**

The network meta-analysis included 23 randomized controlled trials with 16,010 participants, evaluating nine pharmacological interventions ranging from traditional symptomatic therapies to four United States Food and Drug Administration- and National Medical Products Administration-approved disease-modifying therapies, notably anti-amyloid beta monoclonal antibodies. Aducanumab significantly improved ADAS-cog scores compared with placebo (MD -5.97, 95%CI -10.33, −1.61; SUCRA: 93.0%) and demonstrated notable improvements in ADCS-ADL scores (MD 4.99, 95%CI 2.27, 7.72; SUCRA: 98.6%). Memantine ranked highest for neuropsychiatric symptoms (SUCRA: 80.8%). Aducanumab also had the highest SUCRA for CDR-SB (91.5%) and showed moderate superiority in MMSE scores (MD 3.55, 95%CI 1.35, 5.75; SUCRA: 98.2%).

**Conclusion:**

Symptomatic treatments, especially memantine for neuropsychiatric symptoms, remain effective. However, the network meta-analysis indicates that, for patients with mild cognitive impairment or mild Alzheimer’s disease, aducanumab demonstrates the greatest potential for cognitive and clinical improvement (MMSE, ADAS-cog, ADCS-ADL), despite associated risks such as adverse events and amyloid-related imaging abnormalities linked to disease-modifying therapies. Lecanemab provides moderate benefits, while donanemab appears less effective. Thus, clinicians should apply disease-modifying therapies cautiously and individually, carefully balancing potential risks and benefits for each patient.

**Systematic review registration:**

PROSPERO [CRD42025637730], https://www.crd.york.ac.uk/PROSPERO/.

## 1 Introduction

Alzheimer’s disease (AD), identified as the predominant form of dementia in the elderly population, is characterized by the accumulation of extracellular amyloid-beta (Aβ) plaques and the formation of neurofibrillary tangles (NFTs), which result from the hyperphosphorylation of tau protein (Scheltens et al., 2021). According to the World Alzheimer Report 2023^1^, the global prevalence of dementia is projected to increase from 55 million individuals in 2019 to 139 million by 2050, with AD comprising 60–80% of these cases. Notably, AD has become the seventh leading cause of mortality worldwide, highlighting its status as a major public health crisis, further intensified by aging populations and socioeconomic development^2^.

Despite decades of research, therapeutic breakthroughs remain elusive. Current interventions are primarily categorized into two types: symptomatic therapies that temporarily relieve cognitive and behavioral symptoms, and disease-modifying therapies (DMTs), which aim to slow or halt the progression of the disease (Breijyeh and Karaman, 2020). Symptomatic agents, including acetylcholinesterase inhibitors (AChEIs) such as donepezil, rivastigmine, and galantamine, as well as the N-methyl-D-aspartate (NMDA) receptor antagonist memantine, offer limited symptomatic relief without addressing the underlying disease mechanisms. Novel DMTs, primarily focused on targeting Aβ pathology through various mechanisms, are generally considered more suitable for individuals with Mild Cognitive Impairment (MCI) or mild to moderate AD. For instance, aducanumab and lecanemab are monoclonal antibodies (MABs) binding to Aβ protofibrils and plaques, while donanemab selectively targets pyroglutamate-modified Aβ (Jucker and Walker, 2023). In contrast, GV-971 (sodium oligomannate) offers a novel multimodal approach by modulating gut-brain axis dysbiosis to reduce neuroinflammation and Aβ deposition. However, its comparative efficacy, safety, and clinical applicability compared to traditional therapies are still debated. DMTs have the potential to revolutionize clinical practice by impacting treatment strategies, diagnostic methods, and efficacy monitoring. The introduction of anti-amyloid MABs marks a transition toward precision medicine in AD, guided by genetic and biomarker insights (Liu et al., 2025).

Current evidence syntheses regarding AD treatments exhibit fragmentation. Traditional pairwise meta-analyses have typically compared individual agents within specific therapeutic classes. However, there is a paucity of studies that integrate both direct and indirect evidence across diverse interventions. For example, trials investigating DMTs frequently emphasize biomarker outcomes, whereas those evaluating symptomatic treatments often prioritize cognitive scales. This inconsistency poses challenges for cross-comparison, leaving clinicians uncertain about the optimal treatment hierarchies. Network meta-analysis (NMA), which concurrently synthesizes data from multiple interventions, is particularly well-suited to bridge this gap by providing a comprehensive ranking of therapies based on efficacy and safety endpoints. While DMTs such as aducanumab and lecanemab have shown efficacy in Aβ clearance during phase III trials, their cognitive benefits remain in limited and are associated with considerable risks, including amyloid-related imaging abnormalities (ARIA) (Hampel et al., 2023). Conversely, symptomatic treatments provide moderate yet consistent cognitive stabilization, albeit without altering the underlying disease progression. A comprehensive comparative analysis of these therapeutic strategies is currently absent. Safety concerns, such as ARIA associated with anti-amyloid MABs and gastrointestinal side effects linked to AChEIs, underscore the need for an integrated evaluation of their risk–benefit profiles. GV-971, which has demonstrated cognitive improvement in a phase III trial conducted in China (Xiao et al., 2021), though not yet subjected to global regulatory scrutiny, exerts multi-modal effects by modulating gut microbiota, reducing Aβ and tau pathology, and mitigating neuroinflammation, thereby reversing cognitive deficits (Wang et al., 2019). Therefore, this systematic review and NMA aims to assess the cognitive safety and efficacy of 10 distinct interventions: placebo, GV-971, aducanumab, lecanemab, donanemab, donepezil, rivastigmine (both patch and capsule forms), galantamine and memantine. The study intends to provide clinically actionable hierarchies to inform therapeutic decision-making. To our knowledge, this is the first NMA to compare both DMTs and symptomatic therapies with respect to cognitive, functional, and safety outcomes. This analysis is based on a systematic search of five databases and clinical trial registries, addressing critical questions such as whether DMTs offer significant advantages over traditional symptomatic treatments, and whether multimodal approaches, such as combining DMTs with AChEIs, can be justified based on current evidence.

This analysis integrates both indirect and direct evidence from 23 randomized controlled trials (RCTs), aiming to inform clinical practice and outline optimal strategies for the subsequent management of AD.

## 2 Methods

### 2.1 Study design and registration

This NMA protocol was prospectively enrolled with the International Prospective Register of Systematic Reviews (CRD42025637730) and was carried out in alignment with the Preferred Reporting Items for Systematic Reviews and Meta-Analyses (PRISMA) guidelines (Elsman et al., 2024).

### 2.2 Literature search

Following the pertinent guidelines detailed in the Cochrane Handbook for Systematic Reviews of Interventions (Cumpston et al., 2019), two researchers (L-SH and L-Y) independently carried out an extensive search of PubMed, Embase, Cochrane Library, Web of Science, and additional sources from the start until April 2025. The search method was carefully crafted by integrating MeSH terms and free text, specifically designed to align with the unique attributes of every record. The query phrases were systematically classified into three main categories: pharmacological agents, AD, and RCTs. All methods of search were meticulously refined after conducting several initial searches. The drugs included in the search were GV-971, aducanumab, lecanemab, donanemab, donepezil, rivastigmine, galantamine, and memantine. Table 1 outlines a comprehensive search approach for PubMed, serving as an example.

**Table 1:** PubMed search strategy.

Search number	Query
#1	“Alzheimer disease”[MeSH Terms]
#2	“Alzheimer disease*”[Title/Abstract] OR “Alzheimer dementia*”[Title/Abstract] OR “senile dementia”[Title/Abstract] OR “Alzheimer type dementia”[Title/Abstract] OR “primary senile degenerative dementia”[Title/Abstract] OR “primary senile degenerative dementia”[Title/Abstract] OR “Alzheimer syndrome”[Title/Abstract] OR “acute confusional senile dementia”[Title/Abstract]
#3	#1 OR #2
#4	“Donepezil”[Title/Abstract] OR “Aricept”[Title/Abstract] OR “Rivastigmine”[Title/Abstract] OR “Memantine”[Title/Abstract] OR “Aducanumab”[Title/Abstract] OR “aduhelm”[Title/Abstract] OR “BIIB037”[Title/Abstract] OR “Lecanemab”[Title/Abstract] OR “leqembi”[Title/Abstract] OR “BAN2401”[Title/Abstract] OR “GV-971”[Title/Abstract] OR “sodium oligomannate”[Title/Abstract]
#5	#3 AND #4
#6	#5 AND (clinicaltrial [Filter] OR randomizedcontrolledtrial [Filter])

### 2.3 Selection criteria

The investigations considered for inclusion were RCTs issued in English that adhered to the following standards: individuals identified with MCI or AD at any phase of the disease; interventions comprising United States Food and Drug Administration (FDA)-approved treatments, including aducanumab, lecanemab, donanemab, donepezil, rivastigmine (both patch and capsule forms), galantamine, memantine, or National Medical Products Administration (NMPA)-approved GV-971; and trials that compared these anti-AD drugs with placebo or alternative treatments. The reported outcomes included at least one of the following measures: Clinical Dementia Rating-Sum of Boxes (CDR-SB), Mini-Mental State Examination (MMSE), Alzheimer’s Disease Assessment Scale-Cognitive Subscale (ADAS-cog), Alzheimer’s Disease Cooperative Study-Activities of Daily Living (ADCS-ADL), Neuropsychiatric Inventory (NPI), and adverse events (AEs).

The criteria for exclusion were outlined as follows: (i) investigations that were not RCTs, including empirical studies, meta-analyses, systematic reviews, conference abstracts, case reports, editorials, and similar non-primary research; (ii) investigations that did not directly pertain to the research question; and (iii) full-text articles that did not adhere to the PICOS (population, intervention, comparator, outcome, and study design) criteria. In instances where multiple publications stemmed from the same study, only the article with the most comprehensive data and the largest sample size was included. All included articles were selected through consensus among the investigators.

### 2.4 Data extraction

All acquired literature was uploaded into the EndNote software, and duplicate entries were initially eliminated. Two independent researchers (L-SH and L-Y) strictly adhered to the established exclusion and inclusion criteria, evaluating titles and abstracts to exclude irrelevant articles, including reviews, conference papers, and others. Subsequently, a full-text review was conducted for further screening. Data pertinent to the included studies were meticulously extracted, encompassing the year of publication, first author, sample sizes of the two cohorts, mean age, severity of AD, and specifics of the intervention, treatment duration, outcome measures, and details regarding methodological quality. In cases of disagreement between the two researchers, discussions with other team members were held to reach a consensus on whether to include the study.

### 2.5 Data analysis

Analysis of data was conducted utilizing Review Manager version 5.3 (RevMan 5.3) and Stata version 18.0 (Stata 18.0) software. For dichotomous parameters, odds ratios with 95% confidence intervals (CIs) were computed, whereas continuous variables were represented as mean differences (MDs) accompanied by 95% CIs. The quality of the included studies was assessed using RevMan5.3. Heterogeneity testing was performed at a significance level of *p*-value<0.1 and *I*^2^ value>50%. When *p-*value≥0.1 and *I*^2^ value≤50%, studies were considered homogeneous, and a fixed-effect model was applied. Conversely, when *p*-value<0.1 and *I*^2^ value>50%, a considerable degree of variability was presumed, leading to the implementation of a random-effects model (Higgins et al., 2003).

NMA under the frequentist framework was implemented using Stata 18.0. Data preprocessing included generating a network plot and the computation of the Surface Under the Cumulative Ranking Curve (SUCRA) for ranking intervention results. The evaluation of consistency in both direct and indirect comparisons within a closed loop is conducted through the node splitting method, which addresses local inconsistency. When *p*-value ≤ 0.05, local inconsistency is considered significant. A funnel plot adjusted for comparison was subsequently developed to assess potential small-study consequences and publication bias among the investigations included.

## 3 Results

### 3.1 Literature search results

**Figure 1:**
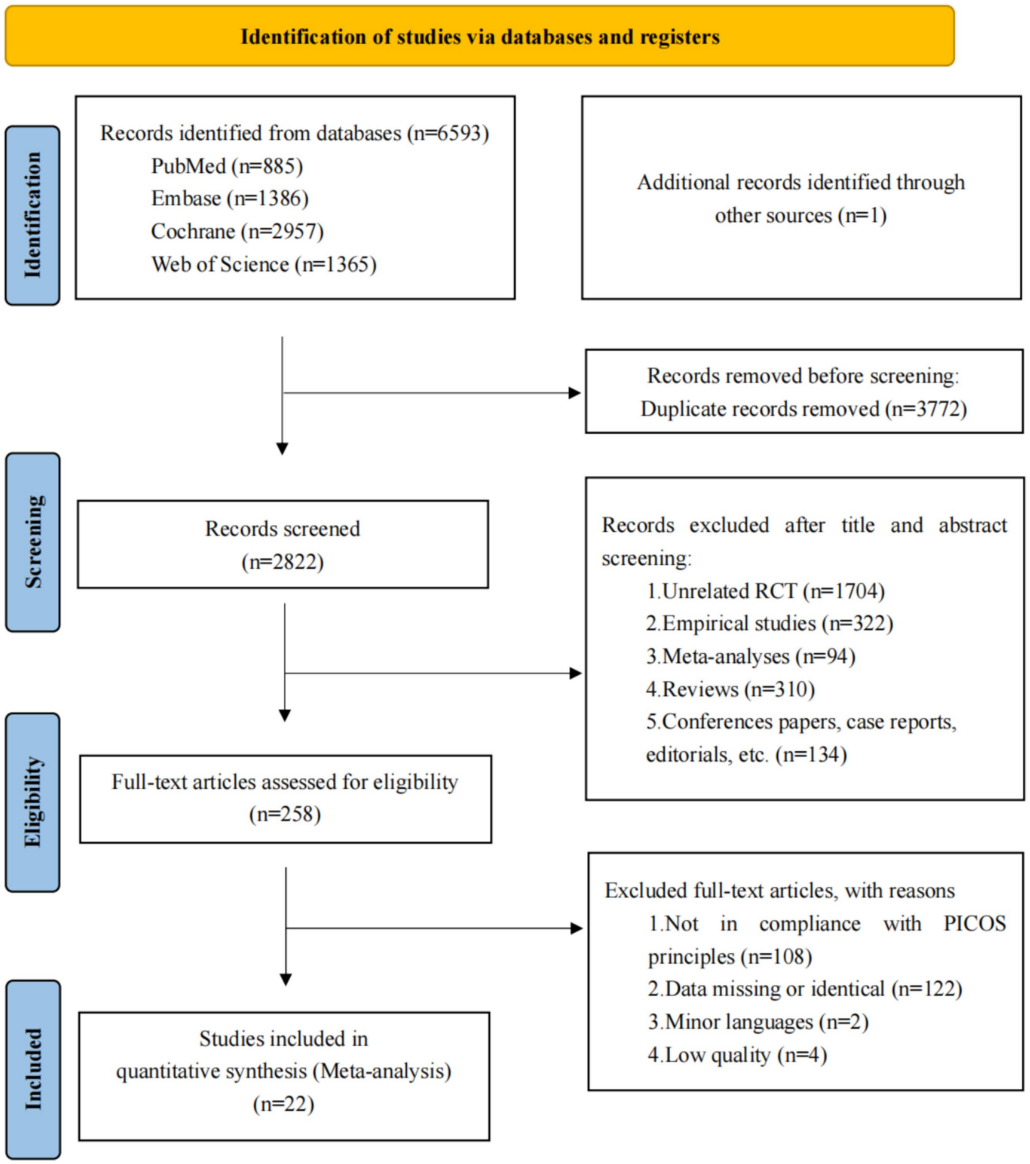
Flow of studies through review. A total of 22 articles were ultimately included, encompassing 23 studies.

A comprehensive dataset of 6,594 articles was sourced from PubMed, Embase, Cochrane Library, Web of Science, and additional sources. After the removal of 3,772 duplicate records, 2,822 articles were excluded following a rigorous review of titles and abstracts. Subsequently, 258 full-text articles were assessed for eligibility, culminating in the inclusion of 22 articles representing 23 studies in the final assessment. The comprehensive screening procedure is depicted in the PRISMA flow chart, as presented in Figure 1.

### 3.2 Eligible studies and patient characteristics

We included RCTs published that met the inclusion criteria. Among these, one study was a three-arm trial, while the others were two-arm trials. The study populations primarily consisted of 4,358 patients with AD who were administered three FDA-approved anti-amyloid MABs (aducanumab, lecanemab, and donanemab). Additionally, 3,970 patients who received AChEIs (donepezil, rivastigmine patch, rivastigmine capsule, and galantamine) were included, as well as 982 patients treated with the oral NMDA receptor antagonist memantine. Moreover, 491 patients treated with GV-971, a brain-gut axis-targeting drug authorized by NMPA in China, were also enrolled. Placebo was the most common control group. The comprehensive baseline characteristics and design elements of the investigations incorporated in this NMA are presented in Table 2.

**Table 2:** Trial features and baseline characteristics of participants for 23 trials included in the network meta-analysis.

Study	Country	Phase	Intervention (dosage)	Sample size		Gender (M/F)		Mean age (year)		Severity of AD	MMSE (baseline)		Follow-up	Outcome
				EG	CG	EG	CG	EG	CG		EG	CG		
Xiao et al. (2021) NCT02293915	China	III	GV-971(900 mg)	408	410	173/235	177/233	69.6 ± 8.12	69.7 ± 8.20	mild-to-moderate AD	19.4 ± 4.4	19.5 ± 4.5	36 weeks	F1, F2, F3, F9
Wang et al. (2020) NCT01453569	China	II	GV-971(900 mg)	83	83	33/50	31/52	70.4 ± 8.5	70.3 ± 8.1	mild-to-moderate AD	18.1 ± 4.4	17.5 ± 4.2	24 weeks	F1, F2, F3, F8, F9
Budd Haeberlein et al. (2022) NCT02484547	20 countries	III	aducanumab low dose (3 mg/kg or 6 mg/kg) high dose (10 mg/kg)	543 547	548	510/553	258/290	70.6 ± 7.4 70.6 ± 7.5	70.8 ± 7.4	MCI or mild AD	26.3 ± 1.7 26.3 ± 1.7	26.4 ± 1.8	78 weeks	F1, F2, F2, F4, F5, F6, F7, F9, F10, F11
Budd Haeberlein et al. (2022) NCT02477800	20 countries	III	aducanumab low dose (3 mg/kg or 6 mg/kg) high dose (10 mg/kg)	547 555	545	526/576	258/287	70.4 ± 7.0 70.0 ± 7.7	69.8 ± 7.7	MCI or mild AD	26.4 ± 1.8 26.4 ± 1.8	26.4 ± 1.7	78 weeks	F1, F2, F3, F4, F5, F6, F7, F9, F10, F11
Swanson et al. (2021) NCT01767311	United States	IIb	lecanemab (10 mg/kg, biweekly)	152	238	88/64	101/137	73	72	MCI or mild AD	25.6 ± 2.4	26.0 ± 2.3	18 months	F1, F5, F9, F10, F11
Sims et al. (2023) NCT04437511	8 countries	III	donanemab (1,400 mg)	low/medium tau:588 combined tau:860	594 876	263/325 367/493	273/321 373/503	74.3 ± 5.7 73.0 ± 6.2	74.3 ± 5.8 73.0 ± 6.2	MCI or mild AD	23.1 ± 3.6 22.4 ± 3.8	22.8 ± 3.8 22.2 ± 3.9	72 weeks	F1, F2, F5, F6, F7, F9, F10, F11
Mintun et al. (2021) NCT03367403	United States	II	donanemab (1,400 mg)	131	126	63/68	61/65	75.0 ± 5.6	75.4 ± 5.4	MCI or mild AD	23.6 ± 3.1	23.7 ± 2.9	76 weeks	F1, F2, F4, F5, F6, F7, F9, F10, F11
Maher-Edwards et al. (2011) NCT00348192	8 countries	II	donepezil (10 mg)	67	61	25/42	18/43	71.1 ± 8.39	71.6 ± 6.72	mild-to-moderate AD	19.2 ± 3.20	18.3 ± 3.36	4 weeks	F1, F3, F9
Peng et al. (2005)	China	III	donepezil (5 mg)	46	43	21/25	19/24	72.6 ± 6.8	71.8 ± 8.2	mild-to-moderate AD	17.8 ± 2.3	18.2 ± 2.7	12 weeks	F2, F4, F5, F9
Zhang et al. (2016) NCT01399125	China		rivastigmine patch [9.5 mg/24 h(10cm^2^)] rivastigmine capsule(12 mg)	248 253		108/140 114/139		70.4 ± 8.02 69.8 ± 8.20		moderate to severe AD	16.0 ± 3.46 16.6 ± 3.08		24 weeks	F1, F2, F3, F4, F9
Grossberg et al. (2011) CENA713D2320	United States		rivastigmine patch [9.5 mg/24 h(10cm^2^)] rivastigmine capsule(12 mg)	278 256	282	94/184 93/163	94/188	73.6 ± 7.5 72.9 ± 8.0	73.9 ± 7.4	moderate to severe AD	16.7 ± 3.0 16.4 ± 3.0	16.4 ± 3.0	24 weeks	F1, F2
Feldman et al. (2007)	6 countries		rivastigmine capsule (2–12 mg)	TID:227 BID:229	222	91/136 98/131	89/133	71.4 ± 7.9 71.0 ± 8.2	71.7 ± 8.7	moderate to severe AD	18.3 ± 4.5 18.8 ± 4.6	18.7 ± 4.6	26 weeks	F1, F4, F9
Karaman et al. (2025)	Turkey		rivastigmine capsule (6–12 mg)	24	20	11/13	9/11	74.11 ± 0.87	73.40 ± 0.90	advanced moderate AD	11.40 ± 0.20	13.20 ± 0.21	12 months	F1, F2, F4, F9
Bullock et al. (2005)	7 countries		rivastigmine capsule (3–12 mg) donepezil(5–10 mg)	495 499		154/341 157/342		75.9 ± 6.6 75.8 ± 6.8		moderately-severe AD	15.1 ± 3.0	15.1 ± 2.9	24 months	F2, F3, F4, F9
Hager et al. (2014) NCT00679627	13 countries		galantamine(8-24 mg)	1,024	1,021	353/671	367/654	73 ± 8.9	73 ± 8.7	mild to moderately severe AD	19.0 ± 4.12	19.0 ± 4.04	24 months	F2, F4, F5, F9
Chu et al. (2007)	China		galantamine(8-24 mg)	33	19	9/24	9/10	78.48 ± 1.61	78.89 ± 1.41	mild-to-moderate AD	14.91 ± 0.60	16.05 ± 0.91	24 months	F1, F2, F3, F4, F9
Hong et al. (2006)	China		galantamine (8–24 mg) donepezil (5–10 mg)	110 108		54/56 49/59		73.3 ± 8.5 74.0 ± 8.4		mild-to-moderate AD	18.8 ± 3.8 17.9 ± 4.1		16 weeks	F1, F2, F3
Brodaty et al. (2005)	5 countries		galantamine (16-24 mg)	326	320	118/208	115/205	76.5 ± 7.77	76.3 ± 8.03	mild-to-moderate AD	17.80 ± 4.14	18.08 ± 4.08	26 weeks	F1, F2, F3
Zhang et al. (2015)	China		memantine (20 mg) donepezil (10 mg)	80 87		31/49 35/52		69.75 ± 8.06 70.13 ± 7.99		mild-to-moderate AD	15.88 ± 4.43 15.53 ± 4.22		12 weeks	F1, F2, F3, F4
Herrmann et al. (2013) NCT00857649	Canada	III	memantine (20 mg)	182	187	77/105	77/110	74.7 ± 7.9	75.1 ± 6.9	moderate-to-severe AD	11.9 ± 3.1	11.8 ± 2.9	24 weeks	F3, F9
Grossberg et al. (2013) NCT00322153	United States		memantine (28 mg)	341	335	97/244	92/243	76.2 ± 8.4	76.8 ± 7.8	moderate-to-severe AD	10.9 ± 2.9	10.6 ± 2.9	24 weeks	F2, F3, F9
Van Dyck et al. (2007)	United States		memantine (20 mg)	178	172	49/129	51/121	78.1 ± 8.2	78.3 ± 7.6	moderate-to-severe AD	10.0 ± 2.8	10.3 ± 3.1	24 weeks	F2, F3, F9
Peskind et al. (2006)	United States		memantine (20 mg)	201	202	80/121	86/116	78.0 ± 7.3	77.0 ± 8.2	mild-to-moderate AD	17.4 ± 3.7	17.2 ± 3.4	24 weeks	F2, F3, F9

Aβ-PET, amyloid-beta positron emission tomography; AD, Alzheimer’s disease; ADAS-cog, Alzheimer’s Disease Assessment Scale-Cognitive Subscale; ADCS-ADL, Alzheimer’s Disease Cooperative Study-Activities of Daily Living; AE, adverse event; ARIA-E, amyloid-related imaging abnormalities characterized by edema and effusion; ARIA-H, amyloid-related imaging abnormalities characterized by cerebral microhemorrhages; CDR-SB, Clinical Dementia Rating scale-Sum of Boxes; CG, control group; EG, experimental group; F1 ADAS-cog, F2 ADCS-ADL, F3 NPI, F4 MMSE, F5 CDR-SB, F6 Aβ-PET, F7 Tau-PET, F8 FDG-PET, F9 AE, F10 ARIA-E, F11 ARIA-H, FDG-PET, fluorodeoxyglucose positron emission tomography; MCI, mild cognitive impairment; MMSE, Mini-Mental State Examination; Tau-PET, Tau positron emission tomography.

### 3.3 Risk of bias

**Figure 2:**
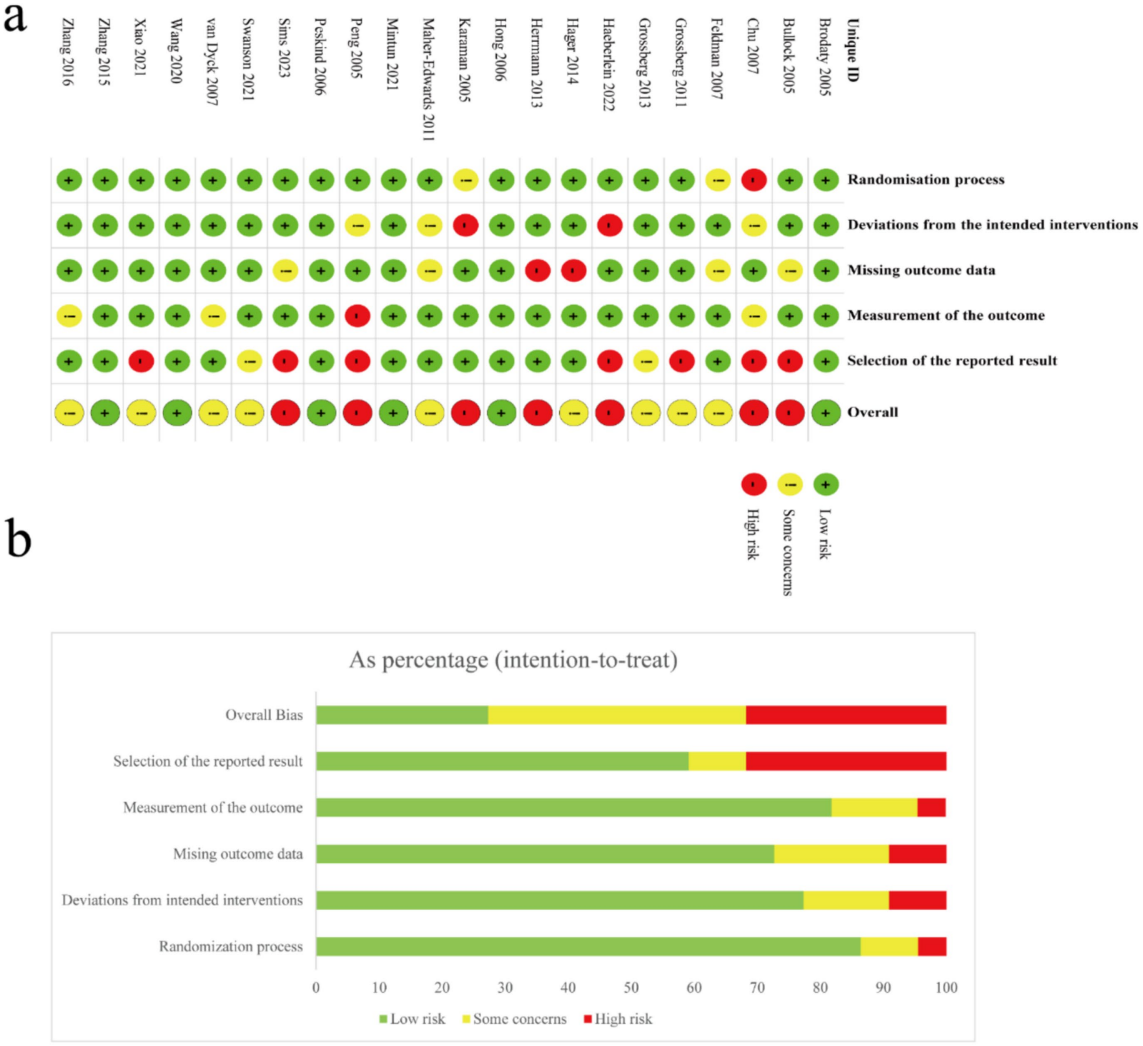
**(a)** Risk of bias graph, and **(b)** risk of bias summary.

The methodological rigor of the 23 included studies was systematically assessed utilizing the Revised Cochrane Risk of Bias Tool version 2.0 (RoB 2.0), focusing on five critical domains: randomization procedures, deviations from intended interventions, completeness of outcome data, objectivity of outcome measurement, and selective reporting. Two independent researchers conducted these assessments, reaching consensus through structured discussions to resolve any initial discrepancies. Of the studies reviewed, 87% had well-documented randomization, earning a “low risk” for selection bias, while 8.7% had insufficient details, and 4.3% had serious flaws. For intervention fidelity, 77.3% adhered well to protocols, 13.6% had moderate issues, and 9.1% showed significant deviations. Two studies faced critical attrition issues, and four had incomplete data documentation. Four studies (17.4%) lacked clear measurement protocols, indicating potential detection bias. No selective outcome reporting was found. Overall, 30.4% of studies were deemed high-risk, and 8.7% had moderate reliability concerns. Visual representations of these risk distributions, including both graphical and tabular summaries, are presented in Figure 2.

## 4 Network meta-analysis

**Figure 3:**
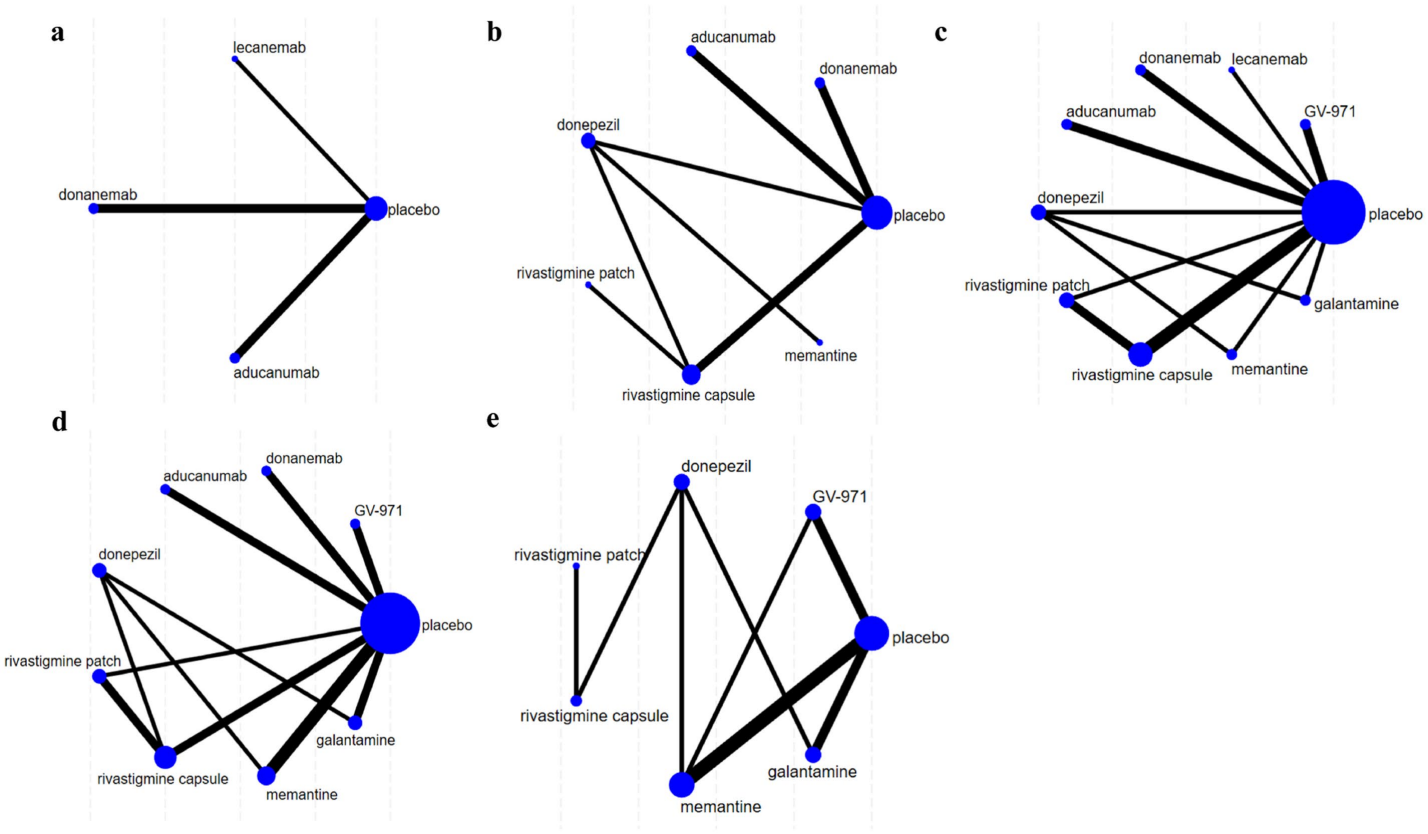
Network diagrams illustrating the comparative effectiveness of various treatments for Alzheimer’s disease (AD). Each node represents a treatment, with the size of the node indicating the number of studies supporting that treatment. The thickness of the lines indicates the strength of evidence comparing treatments. Panels **(a–e)** show different treatment networks, with placebo as a common comparator. **(a)** Clinical Dementia Rating–Sum of Boxes (CDR-SB), **(b)** Mini-Mental State Examination (MMSE), **(c)** Alzheimer’s Disease Assessment Scale–Cognitive Subscale (ADAS-cog), **(d)** Alzheimer’s Disease Cooperative Study-Activities of Daily Living (ADCS-ADL), **(e)** Neuropsychiatric Inventory (NPI).

The NMA map is shown in Figure 3, and detailed NMA results are shown in Supplementary Figure 1.

### 4.1 Cognitive outcomes

#### 4.1.1 Change in CDR-SB

**Figure 4:**
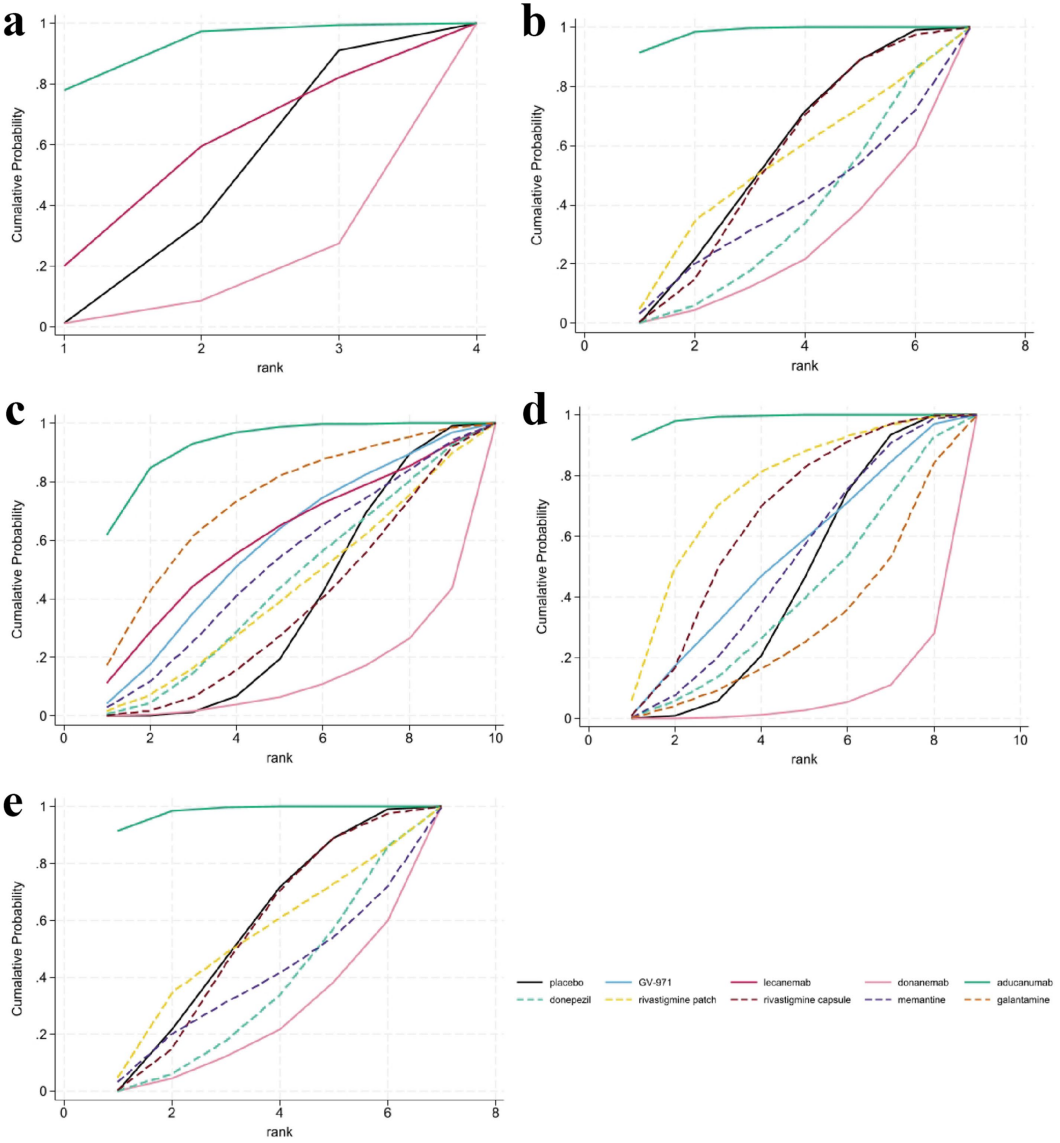
Surface under the cumulative ranking curve (SUCRA) analysis for assessing the relative effectiveness of interventions. SUCRA values, depicted as the area under the curve, provide a probabilistic ranking of interventions, with larger values indicating a greater likelihood of being among the most effective. **(a)** Clinical Dementia Rating-Sum of Boxes (CDR-SB), **(b)** Mini-Mental State Examination (MMSE), **(c)** Alzheimer’s Disease Assessment Scale-Cognitive Subscale (ADAS-cog), **(d)** Alzheimer’s Disease Cooperative Study-Activities of Daily Living (ADCS-ADL), **(e)** Neuropsychiatric Inventory (NPI).

The analysis of CDR-SB scores encompassed data from five RCTs involving three interventions, specifically targeting participants in the mild to moderate stages of AD. The NMA indicated that both aducanumab and lecanemab exhibited significantly greater efficacy than placebo in enhancing CDR-SB scores. Further examination through SUCRA analysis revealed that aducanumab achieved the highest ranking with a score of 91.50%, followed by lecanemab at 53.80%. In contrast, donanemab demonstrated the lowest efficacy, with a SUCRA score of 12.30% (Figure 4a).

#### 4.1.2 Change in MMSE

In the evaluation of changes in the MMSE from baseline, a total of 11 studies involving 7,224 participants were included, with higher scores indicating better cognitive function. Seven interventions were analyzed: aducanumab, donanemab, donepezil, rivastigmine patch, rivastigmine patch, memantine, and placebo. The MD results demonstrated that aducanumab (MD 3.55, 95%CI 1.35, 5.75) significantly improved MMSE scores compared to placebo, with a statistically significant difference. When comparing MMSE scores for the anti-amyloid MABs aducanumab and donanemab, aducanumab (MD 4.94, 95%CI 1.79, 8.08) was found to be superior to donanemab, with statistical significance. The findings from the SUCRA-based probabilistic ranking suggest that aducanumab (SUCRA: 98.20%) is likely the most effective intervention for improving MMSE scores. In contrast, donanemab is ranked lower (SUCRA: 22.80%), indicating a relatively low probability of being the most effective treatment among those evaluated (Figure 4b).

#### 4.1.3 Change in ADAS-cog

The NMA assessed alterations in ADAS-cog scores across 10 treatment regimens, integrating data from 16 RCTs with a total of 8,969 participants, where higher scores denote increased cognitive impairment. The results demonstrated that aducanumab was linked to a statistically significant reduction in ADAS-cog scores compared to placebo (MD -5.97, 95%CI -10.33, −1.61). Aducanumab was identified as the top-ranked intervention, with a SUCRA value of 92.8%, a probability of being the best treatment (PrBest) of 61.8%, and a mean rank of 1.7, indicating it as the most effective option for mitigating AD-related cognitive decline. Conversely, donanemab exhibited the lowest SUCRA value (12.3%) and the highest mean rank (8.9), suggesting minimal therapeutic advantage compared to other treatments. Cumulative probability curves further substantiated the superior efficacy of aducanumab, underscoring its potential as the optimal intervention for enhancing ADAS-cog outcomes (Figure 4c).

### 4.2 Functional and global outcomes

#### 4.2.1 Change in ADCS-ADL

Eighteen articles were reviewed, providing data on changes in the ADCS-ADL scores from baseline, encompassing a total of 10,178 participants across eight interventions: GV-971, aducanumab, donanemab, donepezil, rivastigmine patch, rivastigmine capsule, galantamine, and memantine. Compared to placebo, aducanumab demonstrated a statistically significant improvement in patients’ daily living abilities (MD 4.99, 95%CI 2.27, 7.72), whereas the effects of other treatments did not achieve statistical significance. Notably, the placebo outperformed certain medications, potentially indicating a placebo effect or limitations inherent in the study design. Based on cumulative probability results, aducanumab (SUCRA: 98.60%), rivastigmine patch (SUCRA: 73.00%), and rivastigmine capsule (SUCRA: 63.50%) emerged as the top three interventions for enhancing changes in ADCS-ADL scores, as shown in Figure 4d.

#### 4.2.2 Change in NPI

Results pertaining to changes in NPI score were obtained from 12 RCTs covering six treatment regimens and involving 4,867 participants, where higher figures denoting a greater severity of neurological signs. The six interventions were placebo, GV-971, donepezil, rivastigmine patch, rivastigmine capsule, memantine, galantamine. The NMA facilitated the generation of eight direct or indirect comparisons. The findings indicated that none of the medications exhibited statistically significant differences compared to placebo in improving changes in NPI score. The ranking based on SUCRA values is as follows: memantine (80.8%) > placebo (56.6%) > GV-971 (55.0%) > rivastigmine capsule (47.2%) > rivastigmine patch (43.8%) > donepezil (35.1%) > galantamine (31.0%). The cumulative probability showed that memantine was associated with the greatest benefit on NPI, as shown in Figure 4e.

### 4.3 Safety analysis: adverse events

In clinical trials of anti-amyloid MABs, magnetic resonance imaging (MRI)-detected anomalies, collectively termed ARIAs, have emerged as the principal AEs associated with this therapeutic class. These anomalies predominantly present as ARIA-E (edema/effusion) or ARIA-H (microhemorrhages and hemosiderosis), indicative of vascular inflammatory reactions triggered by amyloid plaque clearance. The disruption of vascular integrity and compromised clearance mechanisms often provoke an immune-mediated inflammatory response within the vessel walls. This transiently compromises vascular stability, leading to the leakage of protein-rich fluid or blood, which manifests as ARIA-E or ARIA-H, respectively (Sperling et al., 2011). Studies have identified the ApoE4 genotype as a notable threat for both ARIA-E and ARIA-H. Individuals carrying the ApoE4 allele exhibit elevated amyloid burden in both the brain parenchyma and vasculature. The enhanced clearance of Aβ facilitated by these therapies, especially in perivascular and interstitial regions, may lead to temporary cerebral amyloid angiopathy and heightened vessel permeability, ultimately promoting the leakage of protein-rich fluid and erythrocytes (Foley and Wilcock, 2024; Filippi et al., 2022). While ARIA-H is often asymptomatic, a subset of ARIA-E cases, particularly at higher dosages, present with symptoms including dizziness, headache, and vomiting. Consequently, careful selection of anti-amyloid MABs, coupled with rigorous pre-treatment, on-treatment, and post-treatment monitoring, is crucial for mitigating AEs. Overall, anti-amyloid MABs demonstrate a favorable safety and tolerability profile. We compared the safety data of three anti-amyloid MABs (aducanumab, lecanemab, and donanemab) in phase III clinical trials. Specific safety indicators and results are detailed in Table 3.

**Table 3:** Comparison of safety data from phase 3 clinical trials of anti-Aβ monoclonal antibodies.

Project	Aducanumab (EMERGE/ENGAGE)	Lecanemab (CLARITY AD)	Donanemab (TRAILBLAZER-ALZ 2)
Safety	EMERGE (High Dose Group) ARIA-E: ApoE4 non-carriers: 18% ApoE4 carriers: 43% ARIA-H: Microhemorrhages: 20% Superficial Siderosis: 33% ENGAGE (High Dose Group): ARIA-E: ApoE4 non-carriers: 23% ApoE4 carriers: 42% ARIA-H: Microhemorrhages: 19% Superficial Siderosis: 16% ENGAGE (Placebo Group): ARIA-H: Microhemorrhages: 7% Superficial Siderosis: 3%	ARIA-E: ApoE4 non-carriers: 5.4% ApoE4 heterozygous carriers: 10.9% ApoE4 homozygous carriers: 32.6% ARIA-H: ApoE4 non-carriers: 11.9% ApoE4 heterozygous carriers: 14% ApoE4 homozygous carriers: 33% Macrohemorrhage: 0.6% Placebo Group: 9%	ARIA-E: ApoE4 non-carriers: 15.7% ApoE4 heterozygous carriers: 22.8% ApoE4 homozygous carriers: 40.6% ARIA-H: Microhemorrhages: 26.8% Superficial Siderosis: 15.7% Intracranial Hemorrhage > 1 cm: 0.4% Placebo Group: 13%

### 4.4 Publication bias test

The funnel plots for all results indicators suggested a balanced distribution of investigations on either side of the central red line, thereby implying a reduced likelihood of publication bias in this review. Details are shown in Supplementary Figure 2.

## 5 Discussion

Over the past three decades, the FDA has authorized the use of several pharmacological treatments for AD, including tacrine, donepezil, rivastigmine, galantamine, and memantine (Ogos et al., 2024). These pharmacological agents primarily target symptomatic relief; however, they exhibit limitations as they predominantly mitigate symptoms without addressing the underlying etiological factors, such as facilitating neuronal regeneration or the clearance of Aβ plaques and hyperphosphorylated tau (Reddi Sree et al., 2025). Professor Jeffery Cummings (Cummings and Fox, 2017) first defined DMTs in 2017 as an emerging therapeutic strategy that targets the core pathophysiological mechanisms of AD to alter its disease trajectory, alleviate symptoms, and delay progression. These strategies encompass the reduction of Aβ deposition, the regulation of tau hyperphosphorylation, the inhibition of neuroinflammation, and the preservation of neuronal survival and synaptic function. As of 2024, the AD drug development pipeline comprises 164 clinical trials evaluating 127 distinct agents. Notably, 75% of these trials are concentrated on DMTs, predominantly involving biologics and small molecules, which target fundamental pathologies such as Aβ plaque formation, tau protein hyperphosphorylation, neuroinflammation, synaptic plasticity dysfunction, and neurotransmitter receptor modulation (Cummings et al., 2024).

Historically, meta analyses on AD drug efficacy and safety centered on symptomatic treatments (Dou et al., 2018; Guo et al., 2020), omitting increasingly prominent DMTs and limiting comprehensive evaluation of treatment strategies. Previous NMA, such as Qiao et al. (2024), focused on anti-amyloid MABs while excluding symptomatic therapies, precluding a holistic assessment. Moreover, several drugs (e.g., bapineuzumab, solanezumab, gantenerumab, crenezumab) in their analysis later failed in clinical trials, thereby calling into question the robustness of the findings. To address these, this NMA comprehensively compares 10 interventions (DMTs and symptomatic treatments) across cognitive, functional, and safety outcomes. DMTs include three anti-amyloid MABs (aducanumab, lecanemab, donanemab) and the gut microbiota-targeting agent GV-971; symptomatic treatments are common clinical agents (e.g., AChEIs, NMDA receptor antagonists).

With the successive approvals of anti-amyloid MABs, DMTs for AD are increasingly prominent, yet their clinical application relies on accurate AD diagnosis. The National Institute on Aging and Alzheimer’s Association (NIA-AA) ATN framework characterizes AD pathophysiology by positive brain Aβ deposition (Jack et al., 2018). Aβ positron emission tomography (PET) imaging, via specific tracers, enables noninvasive localization and quantification of cerebral Aβ plaques, crucial for early diagnosis and therapeutic guidance (Rabinovici et al., 2023). The Centiloid (CL) scale standardizes Aβ PET quantification, ensuring comparability across multicenter studies, tracers, and pipelines, and is widely used in global trials (Klunk et al., 2015). In the CLARITY AD trial, lecanemab demonstrated a reduction in Aβ load by 59.12 CL units, with a mean clearance falling below the 22.99 CL positivity threshold, indicating a potential delay in disease progression of approximately 2 to 3 years. Data from the 2024 Alzheimer’s Association International Conference (AAIC) 3-year follow-up of the CLARITY AD trial revealed sustained improvements in Aβ-PET imaging and the plasma Aβ_42/40_ ratio compared to placebo, underscoring the cumulative benefits of continued lecanemab administration. In the TRAILBLAZER-ALZ 2 trial, the donanemab cohort exhibited an 87.0 CL reduction in brain Aβ levels at week 76, accompanied by reductions of 39.3 and 21.3% in plasma phosphorylated tau217 (p-tau217) and glial fibrillary acidic protein (GFAP), respectively. Regarding aducanumab, Aβ PET imaging indicated 48% amyloid negativity in the EMERGE trial and 31% in the ENGAGE trial; in EMERGE, cerebrospinal fluid (CSF) Aβ_42_ levels increased in a dose-dependent manner, with concomitant decreases in total tau, phosphorylated tau181 (p-tau181), and plasma p-tau181. Similarly, in the ENGAGE trial, CSF Aβ_42_ levels rose dose-dependently with reductions in plasma p-tau181. Nonetheless, the approval of anti-amyloid MABs based solely on the reduction of Aβ plaques is scientifically unjustified, as changes in biomarkers do not consistently correlate with clinical benefits.

Due to the rising attention on early disease stages, DMTs that focus on Aβ are becoming increasingly popular. In the early stages of AD, the disruption of continuous Aβ production and its efficient clearance results in the pathological aggregation of Aβ into misfolded structures. Mabs targeting Aβ are designed to identify and eliminate these harmful aggregates to delay or prevent their formation. Approved anti-amyloid MABs vary in their targets, focusing on different structural regions and aggregation states of Aβ peptides (Kim et al., 2025). Aducanumab, a humanized IgG1 MAB, targets the N-terminus of Aβ with a preference for fibrillary aggregates over monomers, aiding in Aβ clearance via microglial phagocytosis (Dhillon, 2021). Approved by the FDA in 2021 as the first Aβ-targeting therapy, its use is debated. In phase III trials, EMERGE showed a 22% cognitive decline reduction with high doses, while ENGAGE failed its primary endpoint. Both trials noted decreased amyloid plaques and plasma p-tau181, but high doses led to more AEs like ARIA-E, headaches, and brain microhemorrhages. Despite the results of EMERGE, further validation of the efficacy and safety of aducanumab is needed (Budd Haeberlein et al., 2022). Lecanemab is a humanized antibody that crosses the blood–brain barrier to target and bind with Aβ protofibrils, oligomers, and fibrils. Its Fab segment forms complexes with Aβ, while the Fc segment engages microglial receptors to promote phagocytosis, clearing toxic Aβ and reducing neuronal damage. Lecanemab also inhibits Aβ aggregation and slows tau pathology progression (Shi et al., 2022). Approved by the FDA on July 6, 2023, lecanemab showed superior efficacy in slowing cognitive decline compared to placebo in an 18-month phase III trial (van Dyck et al., 2023), with significant changes in CDR-SB scores (*p* < 0.001). It also reduced brain amyloid burden and improved cognitive and functional outcomes. Safety concerns included infusion-related reactions in 26.4% of recipients and ARIA-E in 12.6%, mostly mild to moderate and resolving within four months. Researchers have highlighted potential risks for women and ApoE4 carriers, underscoring the need for careful approval and monitoring (Kurkinen, 2023). Donanemab is a humanized IgG1 MAB that targets N-terminal pyroglutamate-modified Aβ proteins in amyloid plaques, facilitating their removal via microglial phagocytosis. It reduces brain amyloid burden by targeting existing plaques but does not prevent new plaque formation or the growth of existing ones (Buccellato et al., 2023). Approved by the FDA on July 2, 2024, donanemab significantly slowed cognitive and functional decline in Aβ-positive early AD in the phase III TRAILBLAZER-ALZ 2 trial (Dyer, 2024). Nearly half of the early-stage patients exhibited no clinical progression after 1 year, with subgroup analysis revealing a 60% slower decline compared to placebo (Sims et al., 2023).

In this NMA, aducanumab demonstrated superior efficacy in improving cognitive and daily living abilities, aligning with its potent Aβ plaque clearance mechanism. It is worth noting that the initial statements discussed the approvals of aducanumab, lecanemab and donanemab which were based on their ability to reduce Aβ levels, superior to their impact on cognitive function. While the drugs may perform well in Aβ levels, their improvement in patients’ cognitive function in real-world applications may not be as expected (Imbimbo et al., 2023; Richard et al., 2021). Symptomatic agents showed moderate, consistent benefits across cognitive scales, underscoring their role in stabilizing cholinergic and glutamatergic neurotransmission. In terms of mental symptoms, memantine emerged as the top intervention for neuropsychiatric symptoms, likely due to its NMDA receptor modulation mitigating agitation and aggression. Rivastigmine capsule outperformed other AChEI in global clinician assessments, possibly due to dual acetylcholinesterase/butyrylcholinesterase inhibition (Inglis, 2002).

Researchers have found that dysbiosis of the gut microbiota can lead to the accumulation of amino acids such as phenylalanine and isoleucine in the bloodstream. These metabolic products promote the differentiation and proliferation of peripheral immune cells, activating M1-type microglia, which exacerbates neuroinflammation and subsequently accelerates the pathological progression of AD (Wang et al., 2019). GV-971 is an orally given blend of acidic linear oligosaccharides sourced from marine brown algae, created by Shanghai Green Valley Pharmaceuticals, and received first approval in China in November 2019 for use in the management of mild to moderate AD. GV-971 has demonstrated the ability to modify gut microbiota, potentially alleviating the impact of compromised peripheral immunity on AD pathogenesis (Xiao et al., 2021). In a randomized, double-blind, placebo-controlled, multicenter phase III clinical trial conducted in China, GV-971 improved the ADAS-cog scores in patients over a 36-week treatment period, but no significant differences were detected among the drug and placebo groups regarding secondary endpoints, including ADCS-ADL and NPI (Syed, 2020). Several factors may contribute to these results: Firstly, GV-971 early clinical trial data in China have been published, but data from the international multicenter phase III trial are not yet available. The Chinese studies, with small sample sizes and high population heterogeneity, restrict the relevance of the results. Secondly, the ADCS-ADL, an informant-based inventory designed to evaluate everyday activities in AD (Galasko et al., 1997), may experience information bias and measurement bias due to cultural differences. Additionally, the baseline NPI scores were very low (with a mean of 3), limiting the dynamic range necessary to detect measurable improvements. Finally, the limited timeframe of the experiment could have restricted the observation of changes from baseline.

In safety assessments, symptomatic treatments have been used clinically for many years with a relatively low incidence of adverse effects, which are primarily concentrated in the gastrointestinal systems. The side effects of DMTs are primarily a series of discomforts caused by ARIA. The safety profile of aducanumab within the EMERGE and ENGAGE investigations was consistent and in accordance with prior research findings. The most frequently observed AE was ARIA-E, detected via brain MRI. In cases of ARIA-E, severe symptoms have indeed occurred, including intense seizures that required hospitalization. The analysis of the comprehensive safety dataset indicates that the predominant AE observed in the 10 mg/kg aducanumab cohort was ARIA-E, with an incidence rate of 35.2% (362 cases), of which 26.0% (96 cases) presented with related symptoms such as headache (Salloway et al., 2022). As a critical AE associated with anti-amyloid MABs, ARIA-E requires rigorous monitoring and clinical management throughout the treatment course (Budd Haeberlein et al., 2022). In patients receiving donanemab treatment, ARIA-E was observed, with the majority of cases being predominantly asymptomatic. While donanemab demonstrated superiority over placebo in the composite endpoint integrating measures of cognition and activities of daily living, secondary outcomes did not reach statistical significance. Lecanemab is also associated with similar AEs. Additionally, a pharmacovigilance study based on the FDA Adverse Event Reporting System database identified new and unexpected lecanemab-related AEs that were not previously reported in regulatory trials, such as tremors, migraines, pancreatic cancer, et al. Certain patient subgroups, particularly those receiving polypharmacy for AD, as well as those taking aspirin, proton pump inhibitors, statins, antidepressants, or benzodiazepines, may be at a higher risk of experiencing severe AEs (Xing et al., 2025). In light of this, clinicians must place significant emphasis on the aforementioned AEs by closely monitoring the vital signs of participants and systematically assessing potential risks.

Our analysis found that new experimental drugs often show better therapeutic results than established ones, possibly due to optimistic perceptions or biases like selective outcome reporting in early trials. This “newness advantage” suggests inherent biases in DMT trials, needing further validation through IPD-based NMA. *Chin J Intern Med* recently published two consensus documents that systematically outline DMT strategies for AD, integrating the latest clinical evidence and expert insights. The consensus underscores that simultaneously targeting multiple pathological mechanisms, including Aβ, Tau, and neuroinflammation, may enhance therapeutic efficacy. It also advocates for personalized treatment approaches, such as tailoring interventions based on tau pathology burden and ApoE genotype (Neurologist Branch, Chinese Medical Doctor Association; The Expert Group for Expert Consensus on Disease-Modifying Therapy for Alzheimer’s Disease, 2025; Expert Consensus Review Committee on Disease-Modifying Treatments for Early Alzheimer’s Disease, 2025). However, as DMTs transition into clinical practice, several challenges emerge. For instance, community physicians and internists face difficulties in adopting these novel therapies, including limited awareness and experience in managing their side effects. Additionally, there is currently no standardized guidance on whether patients can switch between different anti-amyloid MABs, highlighting the need for further exploration. Consequently, clinicians and patients should critically evaluate the perceived superiority of newly introduced medications by integrating evidence hierarchies and mitigating cognitive biases in therapeutic decision-making.

## 6 Strengths and limitations

This study represents the most recent and largest evidence-based NMA to date, which for the first time evaluates the efficacy of FDA-approved and internationally recognized therapeutic agents for AD over multiple years, encompassing both traditional symptomatic treatments and DMTs. A total of 23 RCTs were included in this analysis, all of which were deemed to be of high quality. Furthermore, the findings of this study are characterized by their authenticity and comprehensiveness. Consequently, this NMA provides comprehensive and rigorous evidence-based recommendations for the treatment and management of patients with AD.

However, there were some limitations to this NMA. Firstly, due to the limited number of head-to-head studies, this NMA primarily relied on indirect estimates, which may affect the accuracy of the results. Secondly, due to different evaluation tools, some scales have poor sensitivity and specificity, which may lead to bias. Thirdly, the inability to obtain sufficient IPD in the RCTs necessitated analysis at a general level, thereby leaving the potential for confounding factors unaddressed. Additionally, the included RCTs vary in participant characteristics, sample size, intervention targets, frequency, and time, which may lead to heterogeneity in results and thus reduce the strength of clinical evidence. Finally, the meta-analysis data were derived solely from publicly available scientific literature, and the publication bias regarding negative results and non-statistical data should be considered, prompting readers to interpret these findings with caution.

## 7 Conclusion

In summary, NMA suggests that aducanumab holds the greatest potential for cognitive and clinical improvements in patients with MCI and early AD, as evidenced by assessments including the MMSE, ADAS-cog, and ADCS-ADL. In contrast, lecanemab provides moderate benefits, while donanemab proves less effective. However, memantine, the traditional symptomatic treatment, remains the preferred option for alleviating neuropsychiatric symptoms in AD patients. The safety profile of DMTs requires further clinical validation. Clinicians must carefully consider biomarker status, disease stage, and safety profiles to optimize personalized treatment strategies for AD. Additionally, owing to the restricted quantity of investigations incorporated in certain interventions, the findings necessitate careful interpretation. Subsequent inquiries ought to prioritize the execution of high-caliber, extensive, and protracted RCTs to substantiate the validity of these findings.

## Data Availability

The original contributions presented in the study are included in the article/Supplementary material, further inquiries can be directed to the corresponding author.

## Footnotes

^1^ https://www.alzint.org/u/world-Alzheimer-report-2024.pdf

^2^ https://www.who.int/news-room/fact-sheets/detail/dementia
