# Supplementary figures and images for: Comparative efficacy and safety of symptomatic therapy and disease-modifying therapy for Alzheimer’s disease: a systematic review and network meta-analysis
Source: Front Neurosci. 2025 Nov 13;19:1656906. doi: 10.3389/fnins.2025.1656906 (PMC12657434; doi:10.3389/fnins.2025.1656906)

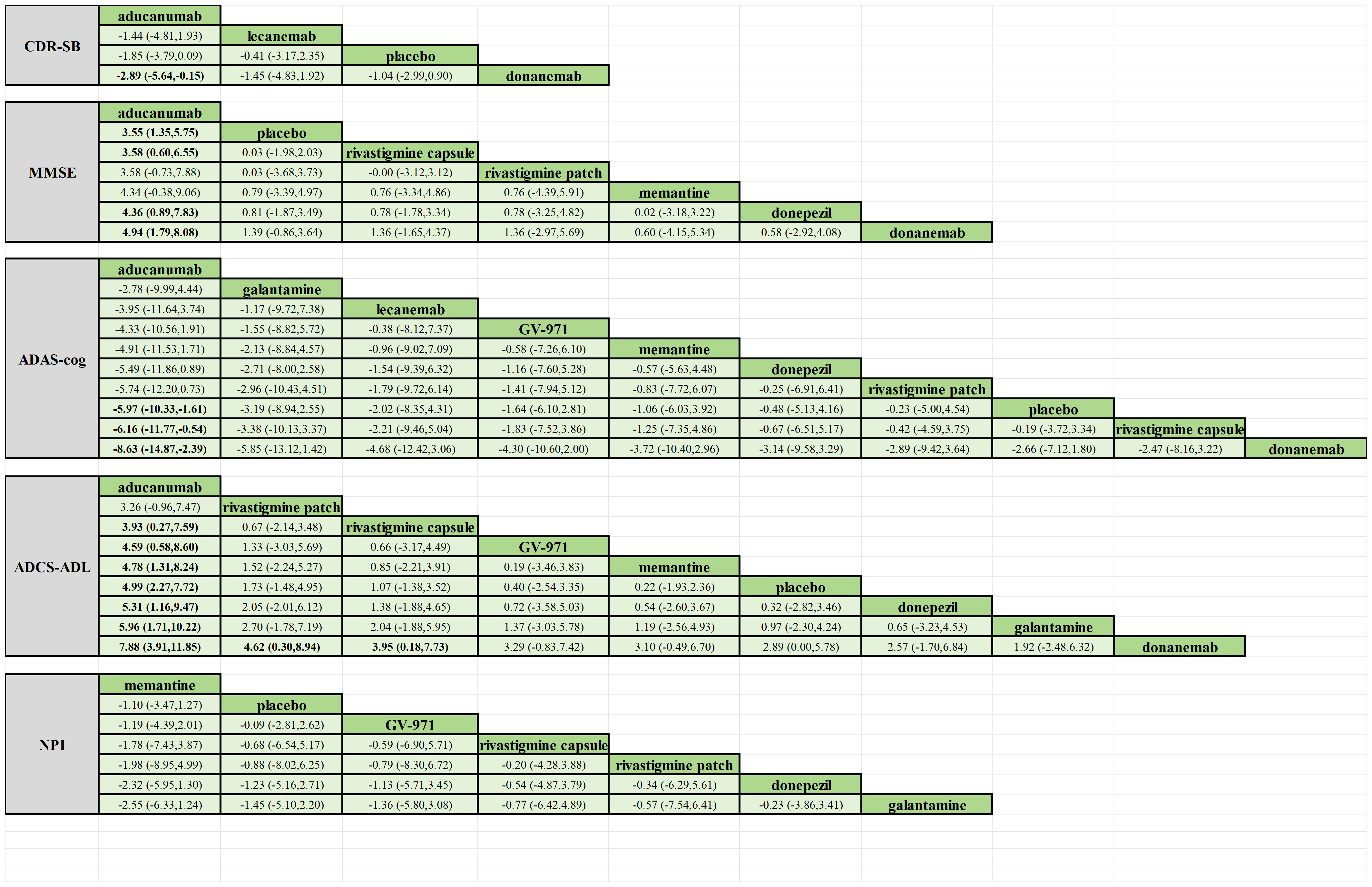

Supplement: Supplementary file 1 [file Image_1.png]

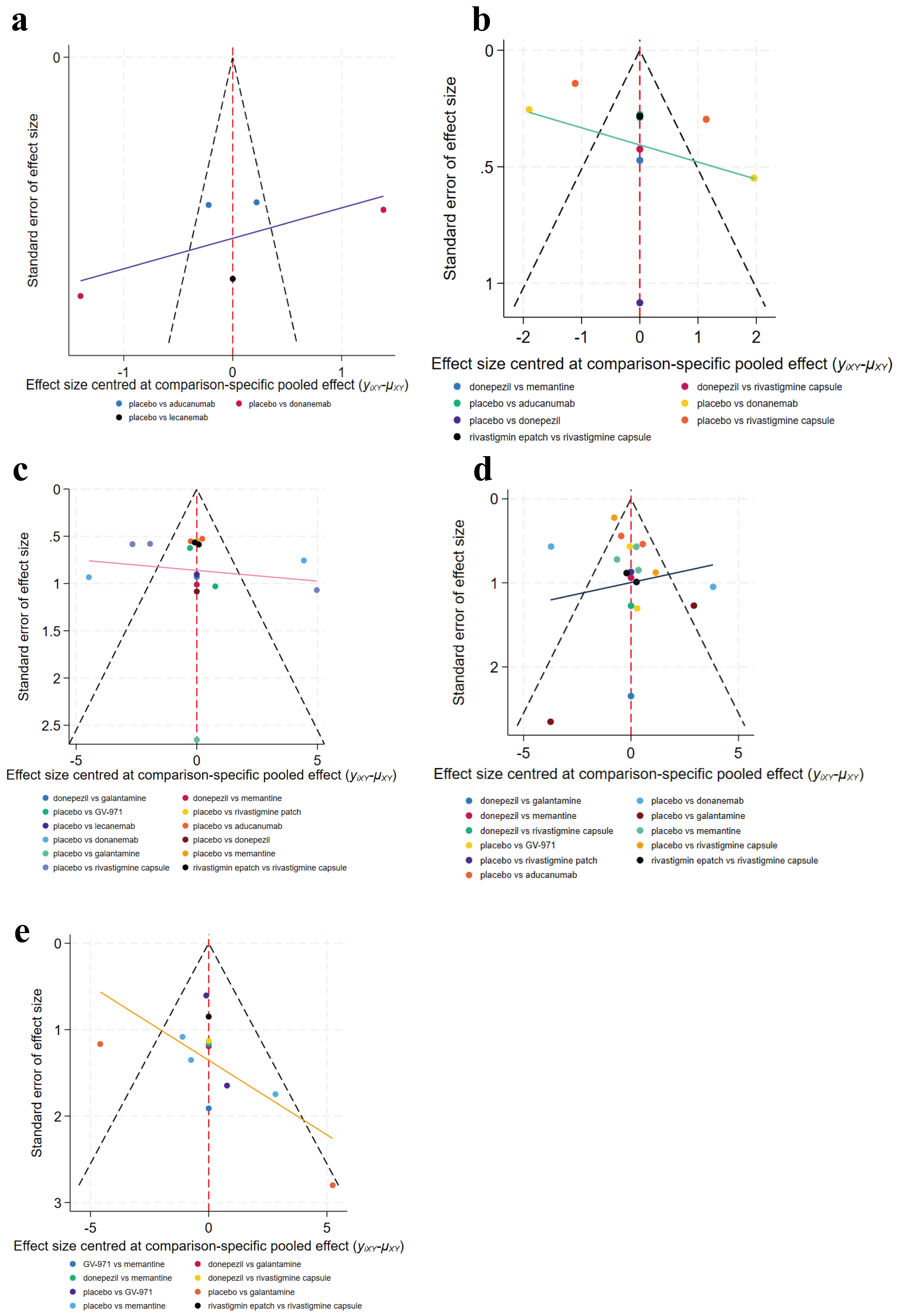

Supplement: Supplementary file 2 [file Image_2.tif]
